# *N*-Centered Chiral Self-Sorting and Supramolecular Helix of Tröger's Base-Based Dimeric Macrocycles in Crystalline State

**DOI:** 10.3389/fchem.2019.00383

**Published:** 2019-05-31

**Authors:** Yuan Chen, Ming Cheng, Benkun Hong, Qian Zhao, Cheng Qian, Juli Jiang, Shuhua Li, Chen Lin, Leyong Wang

**Affiliations:** ^1^Key Laboratory of Mesoscopic Chemistry of MOE, Jiangsu Key Laboratory of Advanced Organic Materials, School of Chemistry and Chemical Engineering, Nanjing University, Nanjing, China; ^2^School of Petrochemical Engineering, Changzhou University, Changzhou, China

**Keywords:** tröger's base, chiral self-sorting, supramolecular helix, gas adsorption, *N*-centered chirality

## Abstract

Three stereoisomers of Tröger's Base-based dimeric macrocycles Trögerophane **1** (**T1**) including one pair of enantiomers (***rac*-T1**) and one meso isomer (***R***_2*N*_***S***_2*N*_**-T1**) were obtained and fully characterized by X-ray analysis. In the crystalline stacking state ***R***_2*N*_***S***_2*N*_**-T1** showed heterochiral self-sorting behavior along *a* axis with cofacial π*-*π stacking interactions, while ***rac*-T1** showed heterochiral self-sorting behavior along *c* axis with slipped π-π stacking interactions, respectively. Meanwhile both of them showed homochiral self-sorting behavior along *b* axis as well as one pair of supramolecular helixes were formed in both cases. All the self-sorting behaviors are controlled by two chiral Tröger's Base units from neighboring molecules. To the best of our knowledge, such chiral self-sorting and supramolecular helixes of *N*-centered chiral superstructures is a rare example. In addition, ***R***_2*N*_***S***_2*N*_**-T1** and ***rac*-T1** demonstrated different adsorption capacities toward the vapor of dichloromethane and acetone, respectively.

## Introduction

Chirality is ubiquitous in the abundant forms of fundamental and crucial processes to create well-order functional structures (Liu et al., 2015; Xing and Zhao, 2018) from natural chiral products amino acid, carbohydrate, nucleic acids, to biomacromolecules of proteins, DNA double helix, to macroscopic systems of chiral crystals, and even to the spiral nebulae of the macroscopic universe.

Chiral self-sorting (Jedrzejewska and Szumna, 2017; Shang et al., 2018) is known as one of self-sorting (Safont-Sempere et al., 2011; Imai et al., 2019) behaviors, in which chirality is one of key differentiating factors for the selectivity of self-assembly. In general, chiral self-sorting is classified as chiral self-recognition or chiral self-discrimination based on the chiral recognization (Chen et al., 2015) by itself or the mirror image of enantiomer, resulting in the formation of homochiral (Makiguchi et al., 2015) or heterochiral species (Yao et al., 2016), respectively. Homochiral self-sorting commonly occurs during the breaking of the symmetry in racemic mixtures, and it is mainly found in (i) conglomerates during crystallization from solution to generate homochirality at the single-crystal scale, (ii) self-assembly on solid surfaces, and (iii) other higher-order functional structures. Compared with homochiral self-sorting, heterochiral systems are mostly relied on the mixture of enantiomers.

Currently, most of chiral self-sorting behaviors occured in the simple molecular system and even in the supramolecular self-assembly (Yashima et al., 2016) were based on *carbon stereogenic factor*. To the best of our knowledge, few studies have been attempted to elucidate chiral self-sorting properties based on *nitrogen stereogenic factor* so far, although *nitrogen stereogenic factor*, particularly *nitrogen stereogenic center* was one of the important sources of chirality (Slater et al., 2016; Feng et al., 2018). In order to understand broadly and deeply chiral behaviors: chiral recognition, chiral amplification, and chiral transmission, it was significant to fabricate chiral system possessing *nitrogen stereogenic factors*.

Along this line of consideration, Tröger's Base (TB) fell in our sights as the candidate. In 1887, Tröger first discovered the reaction of *p*-toluidine with formaldehyde in hydrochloric acid, and the product base was characterized as a white solid with the formula C_17_H_18_N_2_. After research by Spielman, Reed, and other researchers, this base was identified as TB (Dolenský et al., 2012). In TB structure, its V-shaped structure and rigid conformation make it be a useful building block to construct various functional architectures in diverse areas such as catalysis (Du et al., 2010), molecular recognition (Shanmugaraju et al., 2017), optical materials (Neogi et al., 2015), and polymer membranes (Yang et al., 2016). In particular, TB unit is an inherently *C*_2_-symmetric chiral compound as a classical example of *nitrogen stereogenic centers* with two *N*-centered chiral units due to the bridged methylene groups of diazocine nitrogen atoms, which prevents the inversion of the configuration around the stereogenic nitrogen atoms.

Since the birth of supramolecular chemistry, a large number of macrocycles (Cantrill et al., 1999; Ogoshi et al., 2016; Liu et al., 2017; Wu et al., 2017; Li et al., 2019) have been constructed and widely used in molecular machines (Erbas-Cakmak et al., 2015), interlocked structures (Akae et al., 2018), supramolecular catalysis (Blanco et al., 2015; Palma et al., 2017), gas adsorption (Li et al., 2018), adsorptive separation (Jie et al., 2018), and smart materials (Qu et al., 2015; Guo et al., 2018). However, the majority of synthetic macrocycles are bridged from repeating achiral functional units, which limit their applications in the chiral fields. Therefore, the efficient and convenient synthesis of covalent organic macrocycles bearing chiral units, typically with *N*-centered chiral TB units, is needed urgently (Weilandt et al., 2009).

TB-based dimeric macrocycles Trögerophane **1** (**T1**) with bridged oligoethylene glycol (OEG) between two TB units was reported in this work. The rectangular-like **T1** possessed four chiral nitrogen centers, and theoretically, should be a mixture of three stereoisomers: one pair of enantiomers and one meso isomer due to the fixed chirality of TB units (***R***_*N*_, ***R***_*N*_ and ***S***_*N*_, ***S***_*N*_) in the macrocycle. In 1998, Inazu (Brahim et al., 1998) reported the synthesis of **T1** with very low yield (< 3.0%) in more than 10 days and stereoisomers of **T1** were not recognized correctly. In our research, three stereoisomers of **T1**: one pair of enantiomers (***R***_*N*_, ***R***_*N*_, ***R***_*N*_, ***R***_*N*_)**-T1**(denoted as ***R***_4*N*_**-T1**) or ***(S***_*N*_, ***S***_*N*_, ***S***_*N*_, ***S***_*N*_)-T1 (denoted as ***S***_4*N*_-***T1***) and one meso isomer (***R***_*N*_, ***R***_*N*_, ***S***_*N*_, ***S***_*N*_)-**T1**(denoted as ***R***_2*N*_***S***_2*N*_-**T1**) were successfully synthesized, separated and characterized by X-ray analysis undoubtedly for the first time.

More interestingly, the obtained racemate crystals ***rac*-T1** showed the heterochiral self-sorting along *c* axis with slipped π-π stacking interactions and the homochiral self-sorting along *b* axis forming supramolecular *P*/*M* helix by each single enantiomer, respectively. However, either pure enantiomer ***R***_4*N*_-**T1** or ***S***_4*N*_-**T1** could not form supramolecular *P*/*M* helix in crystal state. Surprisingly, **T1** meso isomer, ***R***_2*N*_***S***_2*N*_-**T1**, also showed heterochiral self-sorting behavior along *a* axis with cofacial π-π stacking interactions in crystal state, while homochiral self-sorting behavior was observed along *b* axis and supramolecular *P*/*M* helixes were formed although **T1** meso isomer itself is an achiral molecule. All the self-sorting behaviors occurred between two TB units from neighboring molecules and were controlled by the chirality of Tröger's Base units involved.

## Experimental

### Synthesis and Compound Characterization

**Scheme 1:**
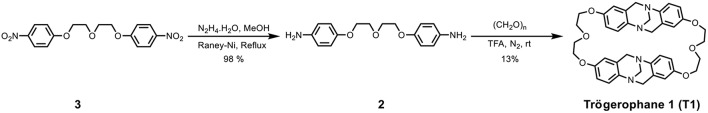
Synthetic route to Trögerophane **1 (T1)**.

As shown in (Scheme 1), **T1** was synthesized from nitroaromatic compound **3**, which was reduced to amine **2**. Then, amine **2** was reacted with 1.5 *equiv*. of paraformaldehyde and trifluoroacetic acid as the solvent under an inert atmosphere over 48 h at the ambient temperature. The resulting reaction mixture was basified (pH >10) using *aq*. NH_3_ and then extracted by dichloromethane, which gave the target dimeric macrocycle **T1** in 13% yield. A combination of ^1^H and ^13^C nuclear magnetic resonance spectroscopy (NMR) and high-resolution mass spectrometry (HRMS) confirmed (Figures S1–S3) the formation of **T1**. The other isolated by-products could be some oligomers or polymers due to the broad peaks observed in the ^1^H NMR spectra.

### Isomer Separation and Circular Dichroism Spectrum

**Figure 1:**
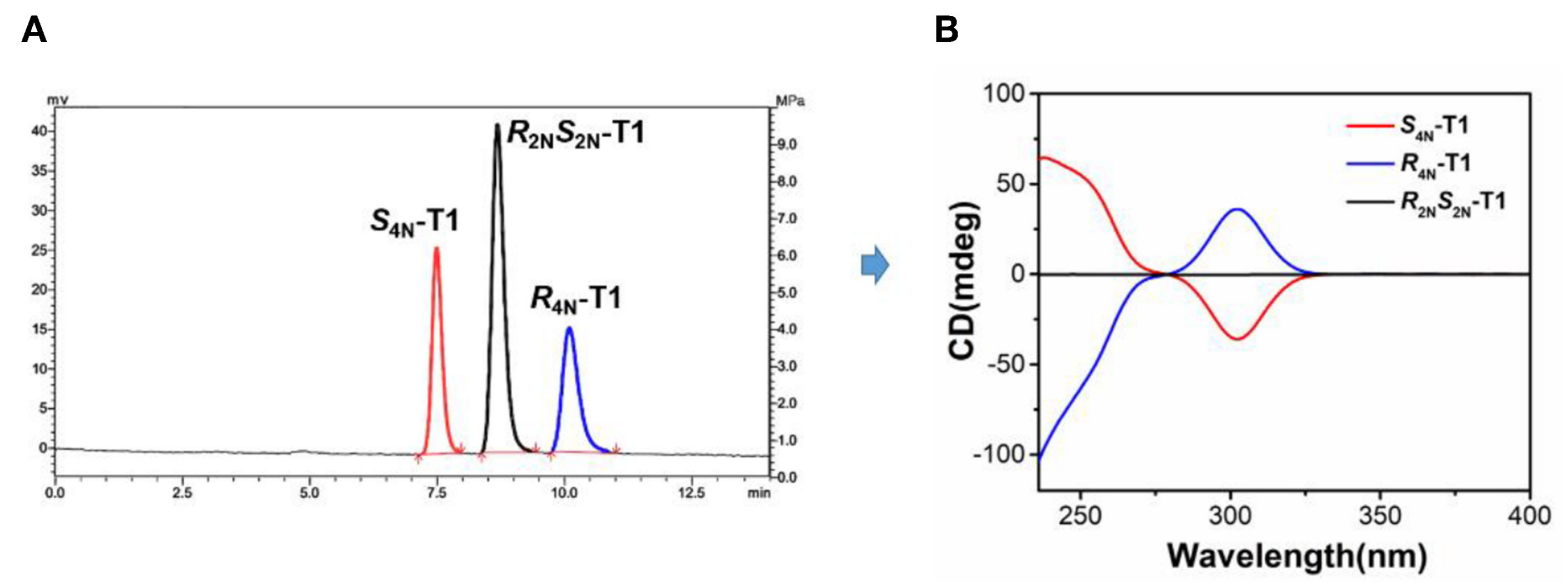
**(A)** Resolution of stereoisomers of **T1** by a chiral HPLC (fractions of ***S***_4*N*_**-T1**, ***R***_2*N*_***S***_2*N*_**-T1**, and ***R***_4*N*_**-T1** from *left* to *right*). **(B)** CD spectra of ***S***_4*N*_**-T1** (0.01 mM*, red*), ***R***_2*N*_***S***_2*N*_**-T1** (0.01 mM*, black*), and ***R***_4*N*_**-T1** (0.01 mM*, blue*) in CH_2_Cl_2_.

The investigation of the stereoisomer structures of **T1** was conducted further by injecting **T1** solution into a chiral HPLC column, and three isolated peaks were observed clearly (Figure 1A). The HPLC spectrum exhibited three single peaks with the mole ratio of 1:2:1, which was well in accord with three types of stereoisomers of **T1**, one pair of enantiomers ***R***_4*N*_-**T1**, ***S***_4*N*_-**T1**, and one meso isomer ***R***_2*N*_***S***_2*N*_-**T1**. Then, these three fractions were collected separately as the single isolated pure stereoisomer for the study of circular dichroism (CD) spectroscopy. The CD spectrum of one of stereoisomers corresponding to the first fraction of HPLC spectrum exhibited a strong negative CD signal at 304 nm, and one of stereoisomers corresponding to the third fraction of HPLC spectrum exhibited a mirror spectrum with positive CD signals at the same wavelength, while another stereoisomer corresponding to the second fraction of HPLC spectrum showed no active Cotton effects in CD spectrum. Experimental results given by HPLC and CD indicated that the second fraction should be assigned to the meso structure (Figure 1B). As a result, **T1** can be confirmed experimentally as the mixture of three stereoisomers with the mole ratio of 1:1:2 of a pair of enantiomers of ***R***_4*N*_-**T1**, ***S***_4*N*_-**T1**, and one meso isomer ***R***_2*N*_***S***_2*N*_-**T1**, respectively.

### Crystal Structure Analysis

**Figure 2:**
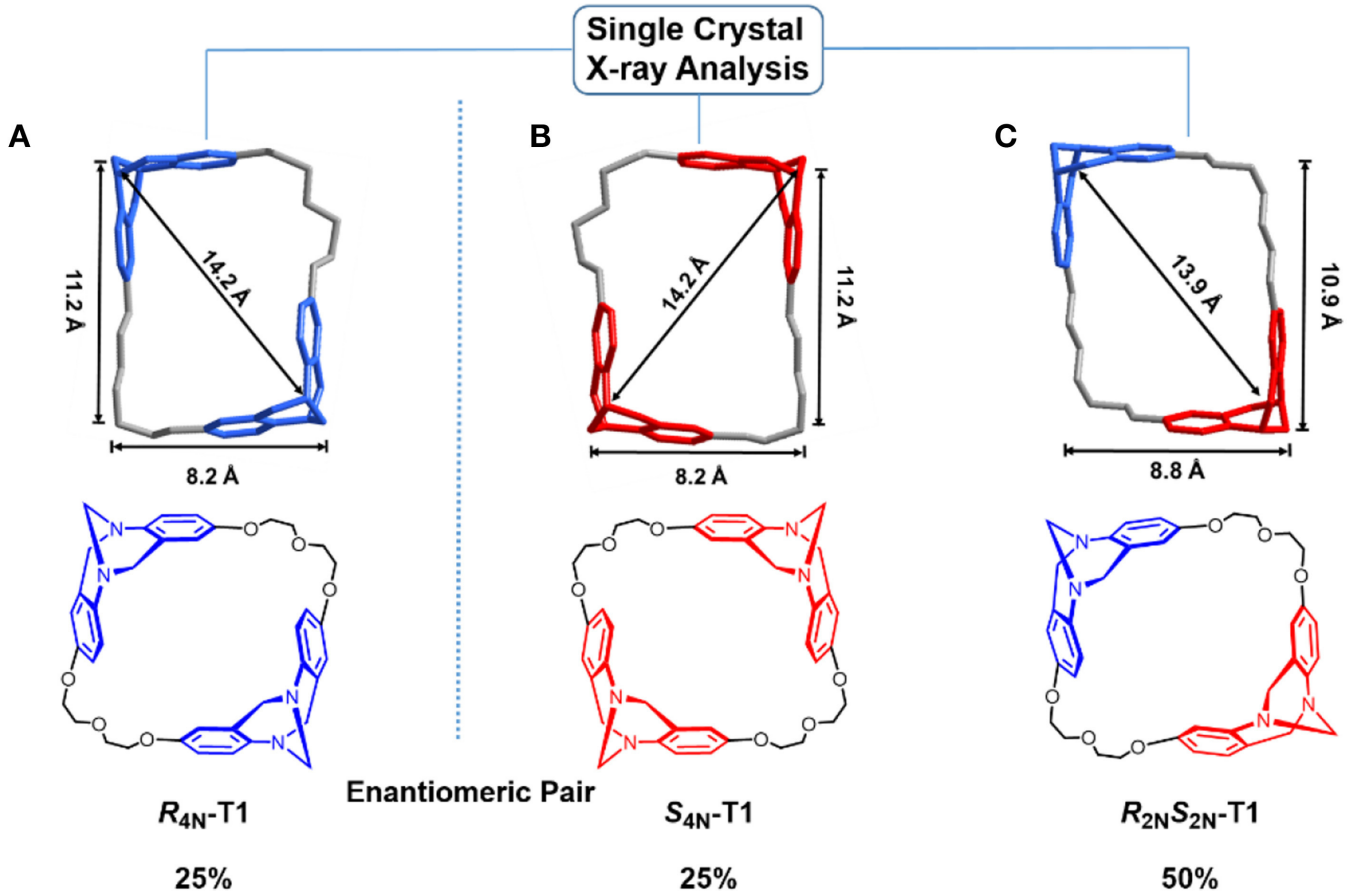
Crystal structures of enantiopure ***R***_4*N*_-**T1 (A)**, enantiopure ***S***_4*N*_-**T1 (B)**, and ***R***_2*N*_S_2*N*_-T1 **(C)** assigned to corresponding chemical structures of stereoisomers. TB units with ***S***_*N*_***S***_*N*_is depicted in red, while TB units with ***R***_*N*_***R***_*N*_ is shown in blue.

Single crystals of ***S***_4*N*_-**T1**, ***R***_2*N*_***S***_2*N*_-**T1**, and ***R***_4*N*_-**T1** suitable for X-ray diffraction were obtained from each isolated pure fraction of the preparative chiral HPLC colunm fortunately (For details see Tables S1–S4). In the crystal of ***R***_2*N*_***S***_2*N*_-**T1**, the cavity size as defined by the distance between two bridged methylene carbons is 13.9 Å in diagonal length, and the distance from the centroid of the benzene rings to the opposite bridged methylene carbon is 10.9 Å and 8.8 Å, respectively. For each crystal of enantiopure ***S***_4*N*_-**T1** or enantiopure ***R***_4*N*_-**T1**, the distance between two bridged methylene carbons is 14.2 Å, with average dimensions of 11.2 × 8.2 Å^2^. As shown in (Figure 2), all the three stereoisomers have rare rectangle-like shapes with four *nitrogen stereogenic centers* and relatively big cavities (>1 nm).

**Figure 3:**
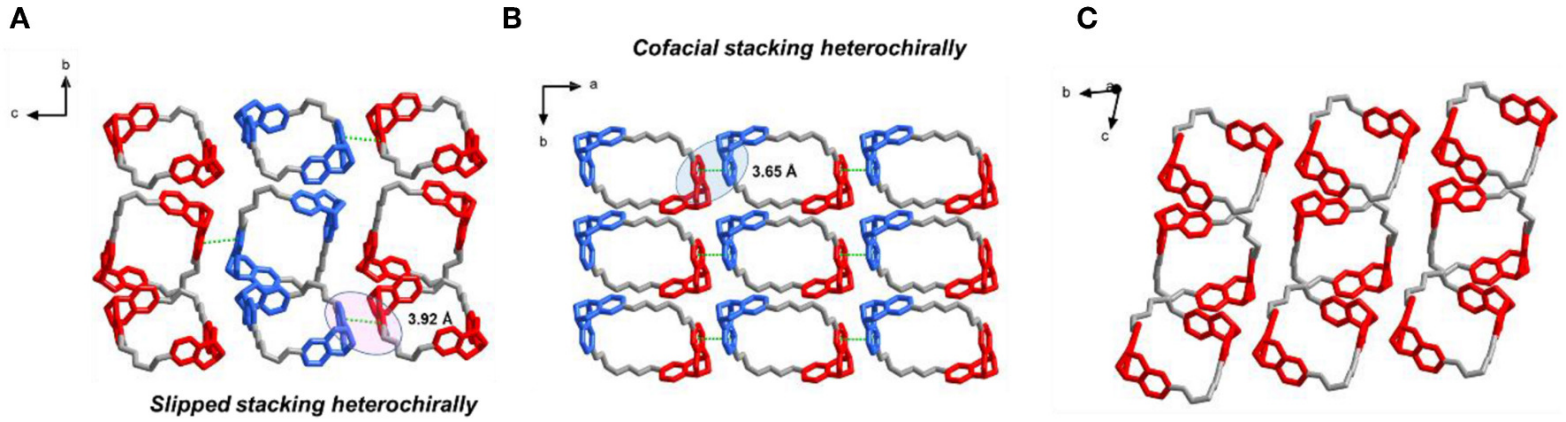
Solid-state (super)structures of ***R***_2*N*_***S***_2*N*_**-T1**, ***rac*-T1**, and ***S***_4*N*_**-T1** obtained from single-crystal X-ray crystallography. **(A)** Slipped π-π stacking between ***R***_4*N*_**-T1** and ***S***_4*N*_**-T1** molecules observed in ***rac*-T1** crystal packing (*left*). **(B)** Cofacial π-π stacking between two ***R***_2*N*_***S***_2*N*_**-T1** molecules observed in crystal packing (*middle*). **(C)** Solid-state (super)structures of ***S***_4*N*_**-T1** molecules observed in crystal packing (*right*). TB units with ***S***_*N*_***S***_*N*_ are depicted in red, while TB units with ***R***_*N*_***R***_*N*_ are shown in blue.

Slow evaporation of a solution of T1 in acetone/diisopropyl ether 1:1 afforded X-ray quality crystals of the racemate. Analysis of the packing of the enantiomers of T1 allowed us to understand how enantiomer segregation takes place. In the crystal of ***rac*-T1**, two neighboring enantiomers stacked *via* a slipped π-π interaction along *c* axis with the distance 3.92 Å, and showed heterochiral self-sorting behaviors (***R***_*N*_***R***_*N*_-*to*-***S***_*N*_*S*_*N*_) between two TB units from neighboring molecules (Figure 3A). Compared with the crystal of ***rac*-T1**, in the crystal of the meso isomer achiral ***R***_2*N*_***S***_2*N*_-**T1** molecules are arranged in such a way that cofacial π-π stacking interactions between neighboring molecules in the *a* direction involve TB subunits of opposite chirality (Figure 3B).

**Figure 4:**
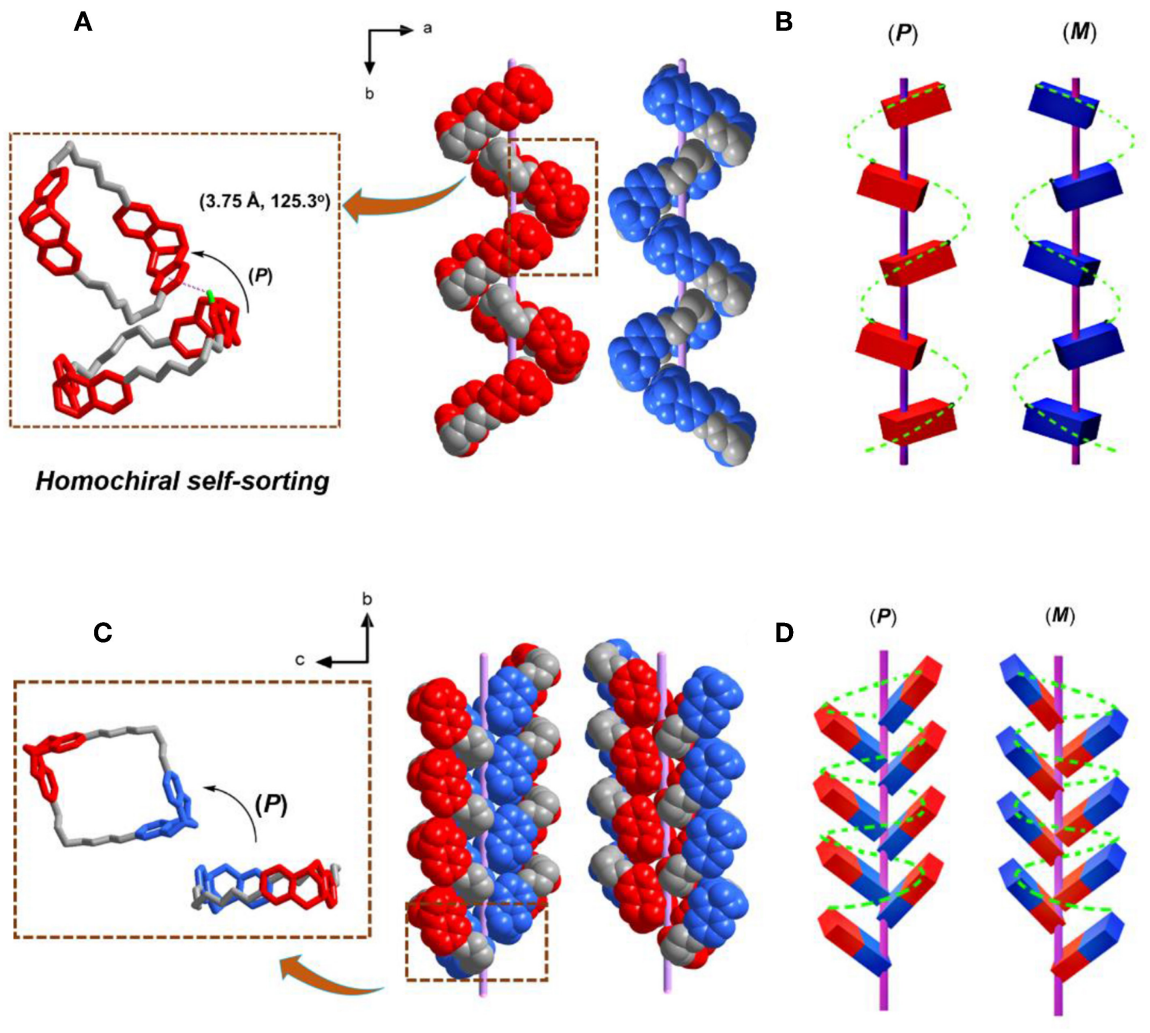
Single-crystal (super)structure of the single helix formed on crystallization of ***rac*-T1** and ***R***_2*N*_***S***_2*N*_**-T1**, respectively. **(A)** Solid-state superstructure of the complementary (*P*) and (*M*)-single helixes formed separately from the enantiomeric ***S***_4*N*_-**-T1** and ***R***_4*N*_**-T1**, respectively. **(B)** The corresponding single helixes of ***S***_4*N*_**-T1** are depicted in red, while the corresponding single helixes of ***R***_4*N*_**-T1** are shown in blue. **(C)** Solid-state superstructure of the complementary (*P*) and *M*-single helixes formed separately from the enantiomeric ***R***_2*N*_***S***_2*N*_**-T1**, **(D)** the corresponding single helixes of ***R***_2*N*_***S***_2*N*_**-T1**. Note: TB units with ***S***_*N*_***S***_*N*_ is depicted in red, while TB units with ***R***_*N*_***R***_*N*_ is shown in blue. For clarity, the solvent molecules inside were also omitted.

Besides the heterochiral self-sorting in the racemate crystal of ***rac*-T1** along *c* axis, the helical chirality was also observed along *b* axis due to the homochiral self-sorting behavior of TB units. In the racemate crystal of ***rac*-T1**, two ***S***_4*N*_-**T1** molecules stacked together by C-H···π interactions (*d*_Cg···*C*_ = 3.75 Å and θ_C−H···*O*_ = 125.3°) between H atom on one of the outwardly tilted phenylene ring and the centroid of another phenylene ring along the *b* axis. The vertical binding energy between two neighboring macrocycles is calculated to be −37.2 kJ/mol (For computational details see Table S5). Then, all ***S***_**4*N***_**-T1** molecules stacked homochirally (***S***_*N*_***S***_*N*_-to-**S**_*N*_**S**_*N*_) along the *b* axis with a rotation of 180°, resulting in a right-handed (*P*) supramolecular single helix. Meanwhile, by the similar self-assembly behaviors, all ***R***_4*N*_-**T1** stacked homochirally (***R***_*N*_***R***_*N*_-*to*-***R***_*N*_***R***_*N*_) along the *b* axis, forming a left-handed (*M*)-single helix. It demonstated that the hands of the single helixes formed in ***rac*-T1** along *b* axis were controlled by homochiral self-sorting behaviors between two chiral TB units (Figures 4A,B). By comparison, in each enantiopure crystal ***R***_4*N*_-**T1** or ***S***_4*N*_-**T1** from the isolated enantiopure solution, no such supramolecular helix was observed (Figure 3C).

In the crystal of ***R***_2*N*_***S***_2*N*_-**T1**, the left/right (M/P)-handed helix along *b* axis was found as well (Figures 4C,D). The chiral two TB units from neighboring molecules along the *b* axis in the case of (***R***_*N*_***R***_*N*_-*to*-***R***_*N*_***R***_*N*_) formed right-handed (*P*) helix and in the case of (***S***_*N*_***S***_*N*_-to-***S***_*N*_***S***_*N*_) formed left-handed (*M*) helix, and the interaction of Van der Waals forces was suggested as the main driving force. The vertical binding energy between two neighboring macrocycles is calculated to be −13.0 kJ/mol (**For computational details see SI**). Remarkably, the achiral meso isomer also shows segregation phenomena in the crystal state, as molecular packing results from interactions between homochiral TB subunits of neighboring achiral macrocycles. Therefore, in both cases, racemic and meso isomer, the supramolecular helical arrangements of the TB macrocycles in the solid state result from preferred intermolecular interactions between homochiral TB subunits.

### Gas Absorption

**Figure 5:**
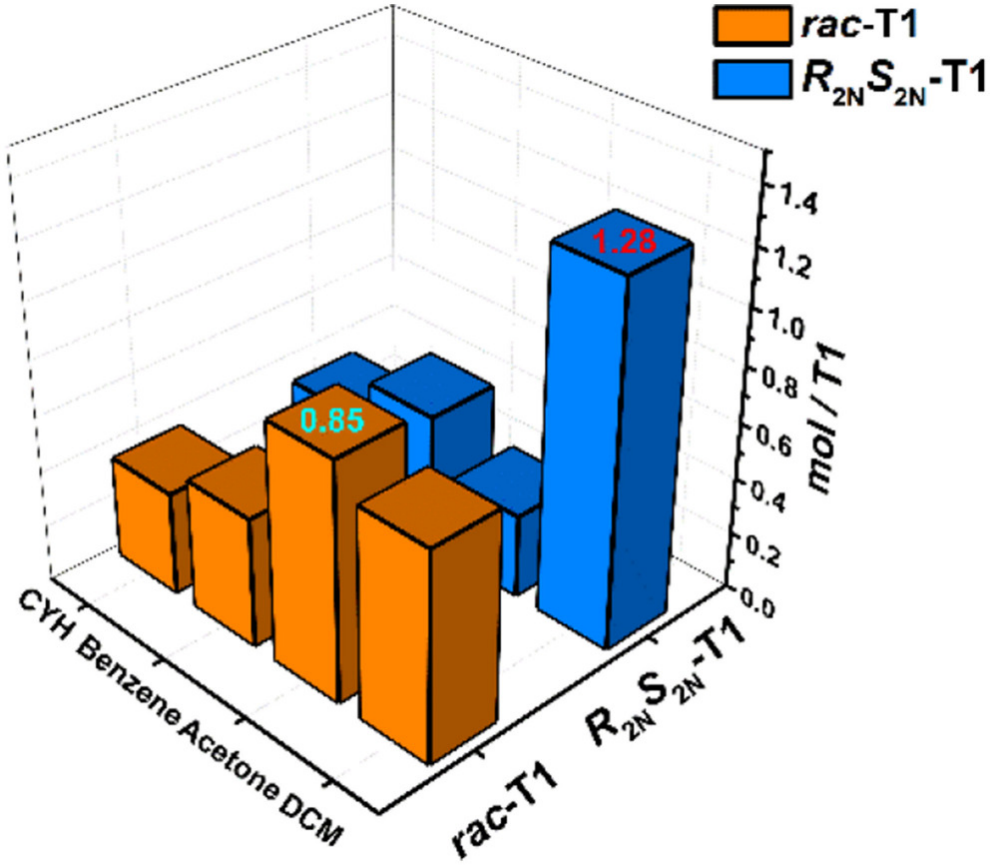
Gas adsorption experiments in ***rac*-T1** or ***R***_2*N*_***S***_2*N*_-T1 with dichloromethane (DCM), acetone, cyclohexane, benzene, respectively.

As shown in Figure 2, three stereoisomers of **T1** have rare rectangle-like shapes and relatively big cavities (>1 nm), so solid-vapor adsorption experiments were carried out to investigate fundamental properties of special cavities of **T1**. The solid powder of ***R***_**2*N***_***S***_**2*N***_**-T1** or ***rac*-T1** was desolvated at 80°C under vacuum, generating the activated solid powder of ***R***_2*N*_***S***_2*N*_-**T1** or ***rac*-T1** (**For details, see SI**). The common solvents dichloromethane (DCM), acetone, benzene, and cyclohexane (CYH) were chosen as vapor sources, and adsorption results were monitored by ^1^H NMR spectroscopy (For details see Figures S4–S11). It was found that the activated powder ***R***_2*N*_***S***_2*N*_-**T1** could selectively adsorb DCM over acetone, benzene, and cyclohexane, and the mole ratio of adsorbed guest molecule: ***R***_2*N*_***S***_2*N*_-**T1** is 1.28, 0.30, 0.48, and 0.33, respectively, where the activated powder ***rac*-T1** could slightly better adsorb acetone over DCM, benzene, and cyclohexane. The mole ratio of adsorbed guest molecule: ***rac*-T1** is 0.85, 0.75, 0.46, and 0.37, respectively (Figure 5).

**Figure 6:**
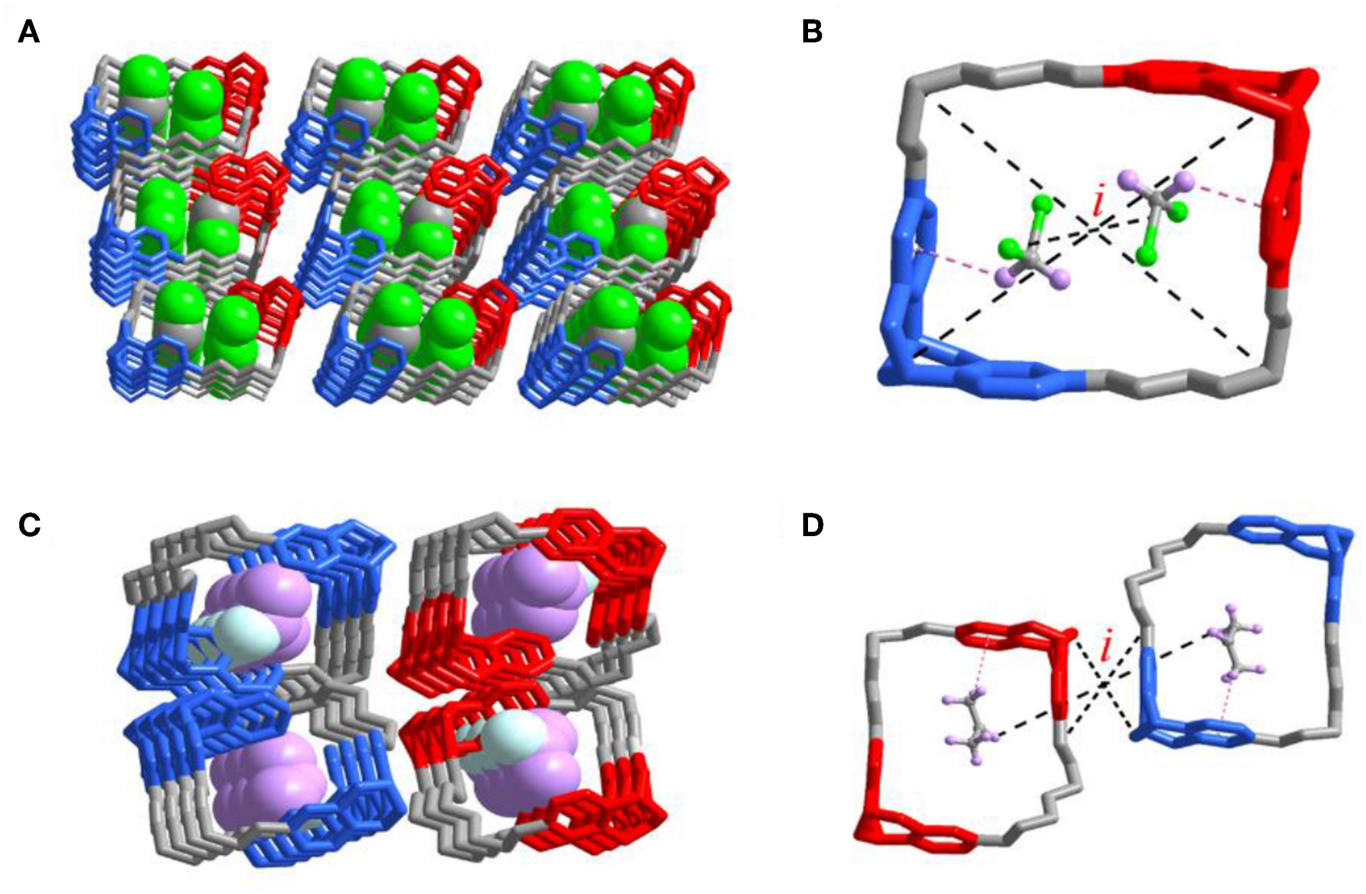
Single-crystal X-ray structures of the complexes **(A)** ***2DCM***@***R***_2*N*_***S***_2*N*_**-T1**, two dichloromethane molecules were bound within the cavity. **(B)** Pink dashed lines indicate the [C-H···π] interactions and ***R***_2*N*_***S***_2*N*_**-T1** encapsulated two dichloromethane molecules. Corresponding guest molecule structural formulas: C, gray; H, pink; Cl, green. **(C)** 2***acetone***@***rac-*T1**, two acetone molecules are bound within the cavity of ***S***_4*N*_**-T1** and ***R***_4*N*_**-T1**, respectively. **(D)** Pink dashed lines indicate the [C-H···π] interactions and ***rac*-T1** encapsulated two acetone molecules. Corresponding guest molecule structural formulas: C, gray; H, pink; O, turquoise. *i:* inversion center.

Although many efforts to get all crystals with different solvents captured inside the cavity failed, the obtained crystal of 2DCM@***R***_2*N*_***S***_2*N*_-**T1** and 2acetone@***rac*-T1** could help us understand the properties of the cavities of ***R***_2*N*_***S***_2*N*_-**T1** and ***rac*-T1** much more. In the crystal of 2DCM@***R***_2*N*_***S***_2*N*_-**T1** (Figure 6A), a 1:2 host-guest complex was formed *via* the favorable C-H···π interaction between DCM and phenylene ring of ***R***_2*N*_***S***_2*N*_-**T1**. Interestingly, an inversion center (*i*) was found inside the crystal of 2DCM@***R***_2*N*_***S***_2*N*_-**T1**. In the racemate crystal of ***rac*-T1**, one acetone molecule was placed in each cavity of ***R***_4*N*_-**T1** or ***S***_4*N*_-**T1** respectively, which was stabilized by C-H···π interactions between acetone and phenylene ring of ***rac*-T1**, forming two 1:1 host-guest complexes. An inversion center was also found in the crystal of 2acetone@***rac*-T1** if 2acetone@***rac*-T1** was treated as a group (Figure 6B). Although the crystal structures were basically in accord with the corresponding selectivity of gas adsorption, further research still need to be conducted to elucidate the intrinsic relation between the formation of the inversion center and the selectivity of vapor adsorption.

## Conclusion

In conclusion, we have synthesized three stereoisomers of Tröger's Base-based dimeric macrocycles **T1** with *four nitrogen stereogenic centers*, which exhibited interesting heterochiral and homochiral self-sorting behaviors between chiral TB units from neighboring molecules along different axis. In the crystal of ***rac*-T1**, two enantiomers stacked *via* a slipped π-π interaction along *c* axis, and showed heterochiral self-sorting behaviors. In the crystal of ***R***_2*N*_***S***_2*N*_-**T1**, neighboring molecules stacked *via* a cofacial π-π stacking along *a* axis, and showed heterochiral self-sorting behaviors as well. In the crystal state, both ***rac*-T1** and ***R***_2*N*_***S***_2*N*_-**T1** showed homochiral self-sorting behaviors and as a consequence, the corresponding (*P*)- and (*M*)- single helixes were formed.

As mentioned above, most of the existing chiral self-sorting behavior was based on *carbon stereogenic factors*. *N*-centered chiral self-sorting example has been rarely reported so far. As the complementary of *carbon stereogenic factors*, our research extends the span and scope of chiral self-sorting behaviors, and paves a way to understand broadly the chirality between different chiral species in different scales.

## Data Availability

CCDC 1888534 (S4N-T1), 1888548 (R4N-T1), 1888547 (R2NS2N-T1) and 1822570 (rac-T1) contain the supplementary crystallographic data for this paper. These data can be obtained free of charge from The Cambridge Crystallographic Data Centre.

## Author Contributions

JJ and CL conceived and designed the study. YC conducted the synthetic experiments, and BH, SL conducted the DFT calculations. All of authors analyzed and interpreted the data. JJ, CL, and LW wrote and revised the manuscript.

### Conflict of Interest Statement

The authors declare that the research was conducted in the absence of any commercial or financial relationships that could be constructed as a potential conflict of interest.
