# Supplementary material for: N-Centered Chiral Self-Sorting and Supramolecular Helix of Tröger's Base-Based Dimeric Macrocycles in Crystalline State
Source: Front Chem. 2019 May 31;7:383. doi: 10.3389/fchem.2019.00383 (PMC6555195; doi:10.3389/fchem.2019.00383)
Supplement: Supplementary file 1 [file Data_Sheet_1.pdf]

Supplementary Material

Article Title *N*-Centered Chiral Self-sorting and Supramolecular Helix of Tröger's Base-based Dimeric Macrocycles in Crystalline State

Yuan Chen[1], Ming Cheng[1], Benkun Hong[1], Qian Zhao[1], Cheng Qian[1], Juli Jiang[1*], Shuhua Li[1], Chen Lin[1*] and Leyong Wang[1,2]

*** Correspondence:**

Juli Jiang: jjl@nju.edu.cn

Chen Lin: linchen@nju.edu.cn

Experimental Procedures

1. General information

All reactions were performed in air atmosphere unless otherwise stated. All reagents and solvents, unless otherwise indicated, were obtained from commercial sources. Melting points (M.p.) were determined using a Focus X-4 apparatus (made in China) and were not corrected. All yields were given as isolated yields. NMR spectra were recorded on a Bruker DPX 400 MHz spectrometer with internal standard tetramethylsilane (TMS) and solvent signals as internal references at 298 K. High-resolution electrospray ionization mass spectra (HR-ESI-MS) were recorded on an Agilent 6540Q-TOF LCMS equipped with an electrospray ionization (ESI) probe operating in positive-ion mode with direct infusion. A preparative Chiralpak IG IG00CE-UC011) column was used for separation of enantiomers. Circular dichroism spectra were measured on a JASCO J-810 ctrometer using a quartz cuvette of 1 cm path length.

2. Synthesis of Trögerophane 1 (T1)

4,4'-((oxybis(ethane-2,1-diyl))bis(oxy))bis(nitrobenzene) **3** and 4,4'-((oxybis(ethane-2,1-diyl))bis(oxy))dianiline **2** were synthesized according to literature procedures (Brahim, 1998).

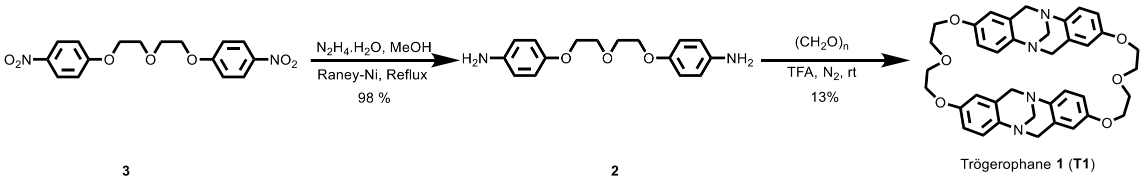

Scheme S1. Synthetic route to **Trögerophane 1 (T1)**

A single-necked flask equipped with a magnetic stirrer was charged with 4,4'-((oxybis(ethane-2,1-diyl))bis(oxy))dianiline **2** (5.76 g, 20.0 mmol) and triformaldehyde (7.21 g, 80.0 mmol) under nitrogen at 0 ˚C, trifluoroacetic acid (500 ml) was slowly added by syringe at 0 ˚C, and then the mixture was stirred at room temperature for 48 hours. Then the reaction was quenched by addition of water (200 mL) after neutralization with aq. NH_3 to pH = 6.5, the mixture was extracted by CH_2Cl_2 (3 × 100 mL), and the organic phase was dried with

anhydrous Na_2SO_4. After filtration and rotary evaporation, the product was purified by column chromatography on silica gel (eluent: 1/50, *v/v*, methanol : dichloromethane gradually changing to 1/25) to give **T1** as a white powder (0.84 g, 1.3 mmol). The ^{1}H NMR matches the data reported in the literature (Brahim, 1998). M.p. (***R$_{4N}$*** or ***S$_{4N}$*** 145-146 °C, ***R$_{2N}$S$_{2N}$*-T1** > 250 °C). ^{1}H NMR (400 MHz, $CDCl_3$) δ = 7.00 – 6.97 (m, 4H), 6.71 – 6.67 (m, 4H), 6.37 – 6.35 (m, 4H), 4.59 (d, J = 16.4 Hz, 4H), 4.28 (d, J = 16.4 Hz, 4H), 4.00 – 3.92 (m, 12H), 3.83 – 3.75 (m, 8H) ppm. ^{13}C NMR (100 MHz, $CDCl_3$): 155.3, 140.5, 128.4, 125.7, 114.4, 115.6, 69.8, 67.7, 67.4, 59.0 ppm. HRMS (ESI-TOF) *m/z*: calcd. for $[C_{38}H_{41}N_4O_6 + H]^+$ 649.3021; found: 649.3016.

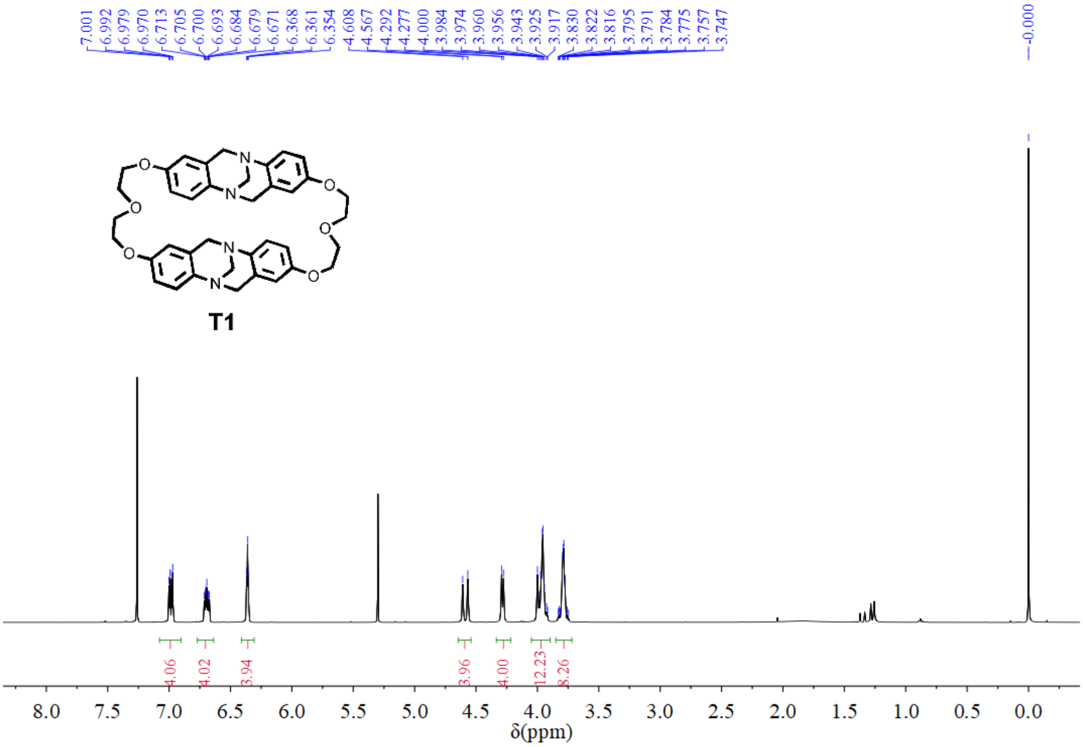

Figure S1. ^{1}H NMR spectrum of (400 MHz, $CDCl_3$, 298 K) **Trögerophane 1 (T1)**.

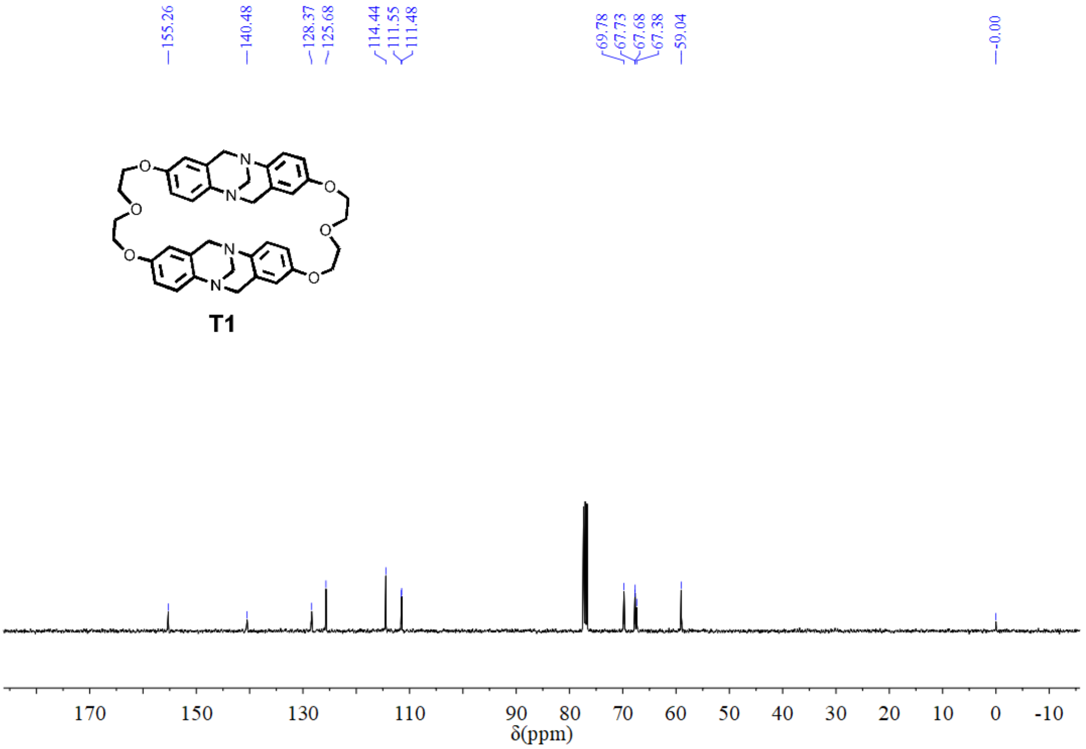

Figure S2. ^{13}C NMR spectrum (100 MHz, $CDCl_3$, 298 K) of **Trögerophane 1 (T1).**

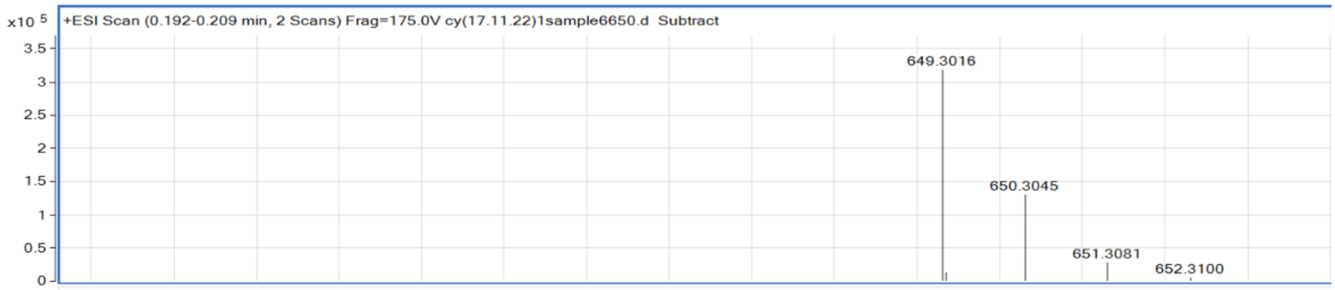

Figure S3. High Resolution ESI-MS of **Trögerophane 1 (T1)** with the parent ion $[M + H]^+$ at 649.3016, corresponding to $C_{38}H_{41}N_4O_6$ with the calculated m/z = 649.3021.

3. Single-Component Adsorption Experiment in *rac-T1* or *R2NS2N*-T1

Activated powder ***rac*-T1** and ***R2NS2N*-T1** was dried under vacuum at 80 °C overnight. For each single-component ***rac*-T1** or ***R2NS2N*-T1** adsorption experiment, an open 5 mL vial containing 0.020 g of solvent-free ***rac*-T1** or ***R2NS2N*-T1** adsorbent was placed in a sealed 20 mL vial containing 5 mL of dichloromethane, acetone, cyclohexane, and benzene, respectively. After adsorption of solvents vapor for 10 hours, and measuring the molar ratio of dichloromethane, acetone, cyclohexane, benzene to ***rac*-T1** or ***R2NS2N*-T1** by ^{1}H NMR was calculated, respectively.

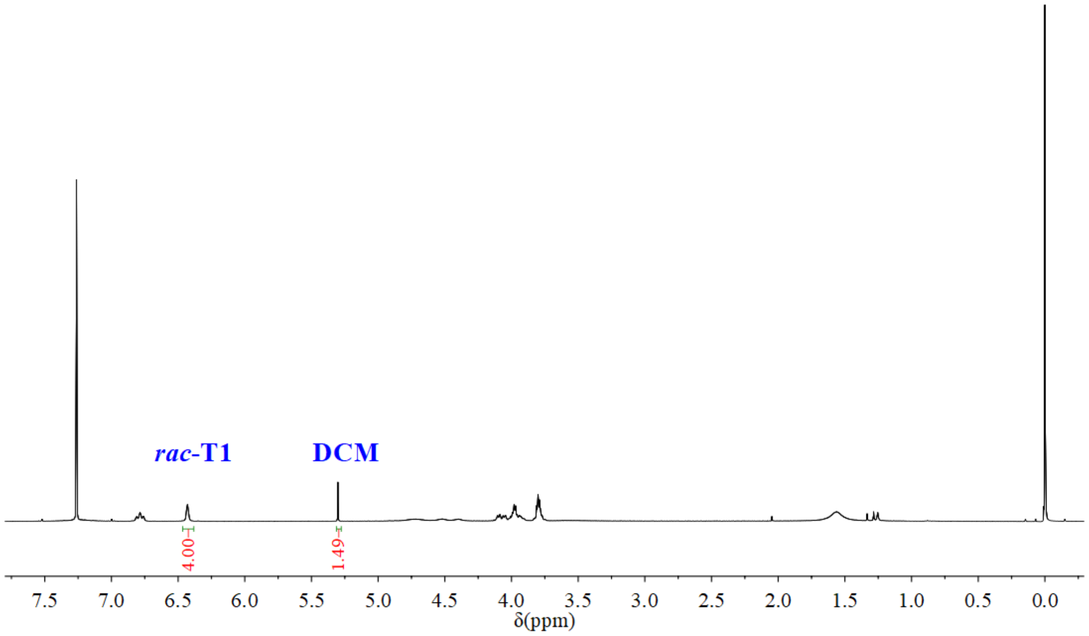

Figure S4. ^{1}H NMR spectrum (400 MHz, $CDCl_3$, 293 K) of ***rac*-T1** after adsorption of dichloromethane vapor for 10 hours.

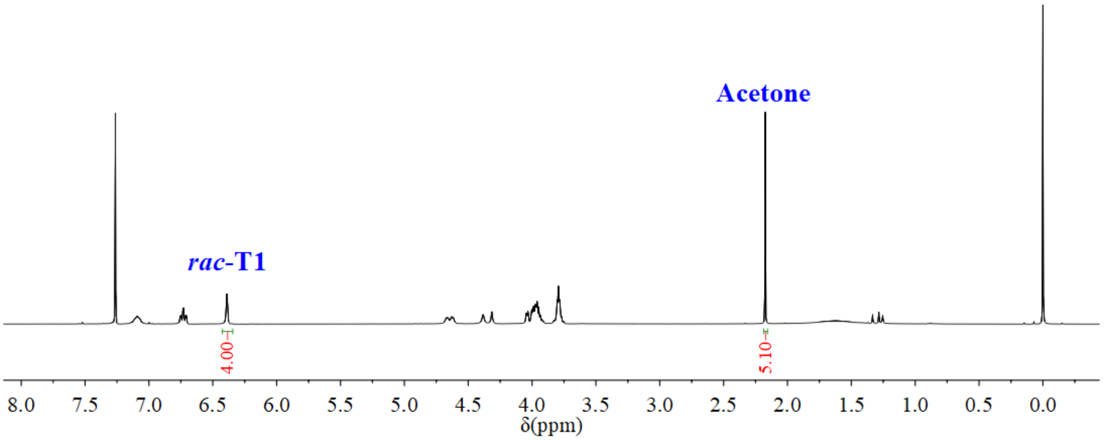

Figure S5. ^{1}H NMR spectrum (400 MHz, $CDCl_3$, 293 K) of ***rac*-T1** after adsorption of acetone vapor for 10 hours.

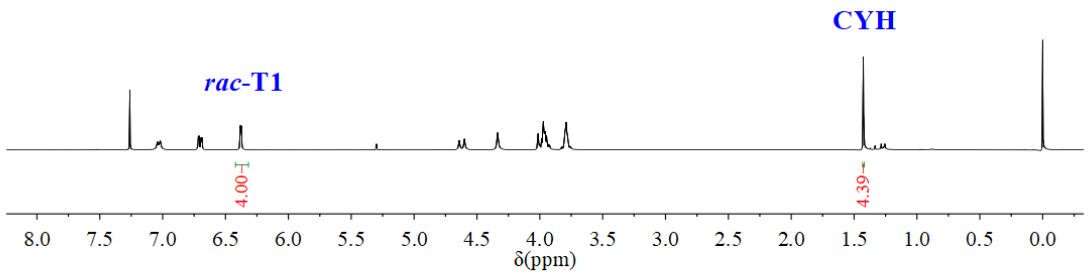

Figure S6. ^{1}H NMR spectrum (400 MHz, $CDCl_3$, 293 K) of ***rac-T1*** after adsorption of cyclohexane vapor for 10 hours.

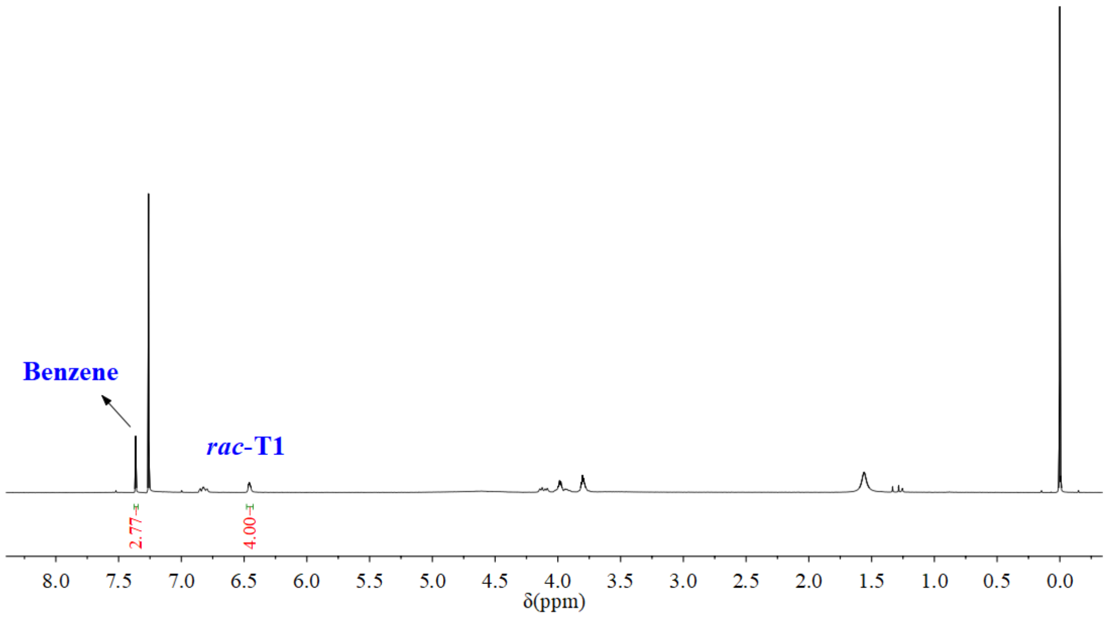

Figure S7. ^{1}H NMR spectrum (400 MHz, $CDCl_3$, 293 K) of ***rac-T1*** after adsorption of benzene vapor for 10 hours.

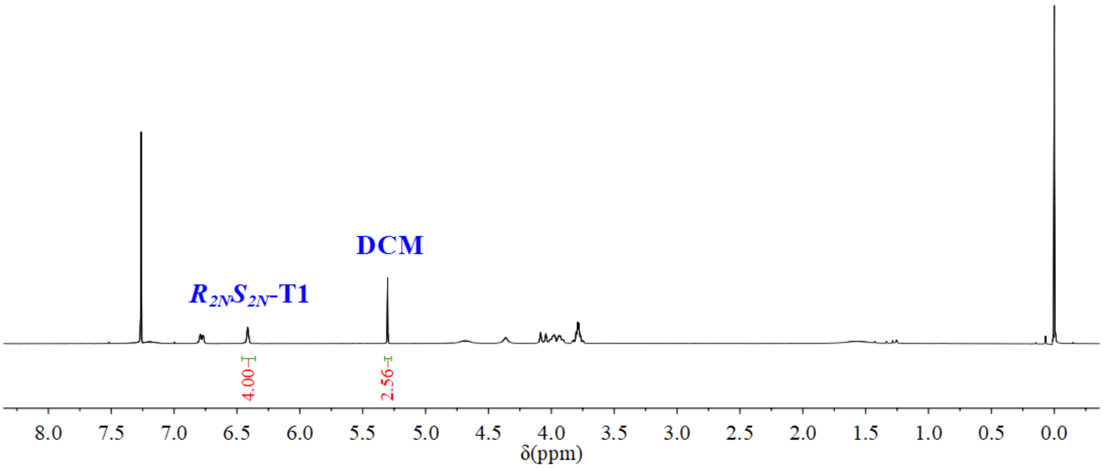

Figure S8. ^{1}H NMR spectrum (400 MHz, $CDCl_3$, 293 K) of ***R***

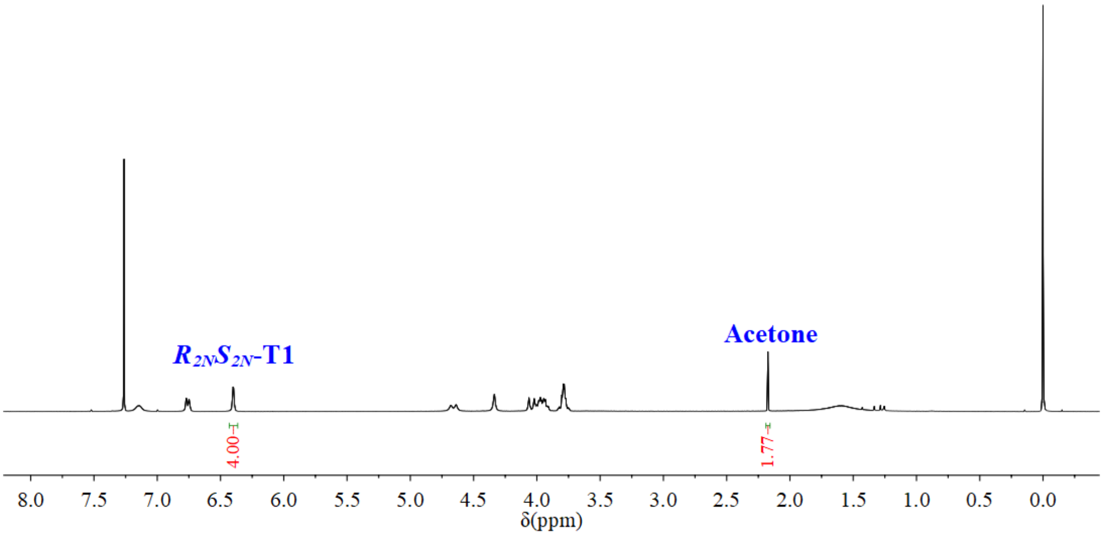

Figure S9. ^{1}H NMR spectrum (400 MHz, $CDCl_3$, 293 K) of $\boldsymbol{R_{2N}S_{2N}}$**-T1** after adsorption of acetone vapor for 10 hours.

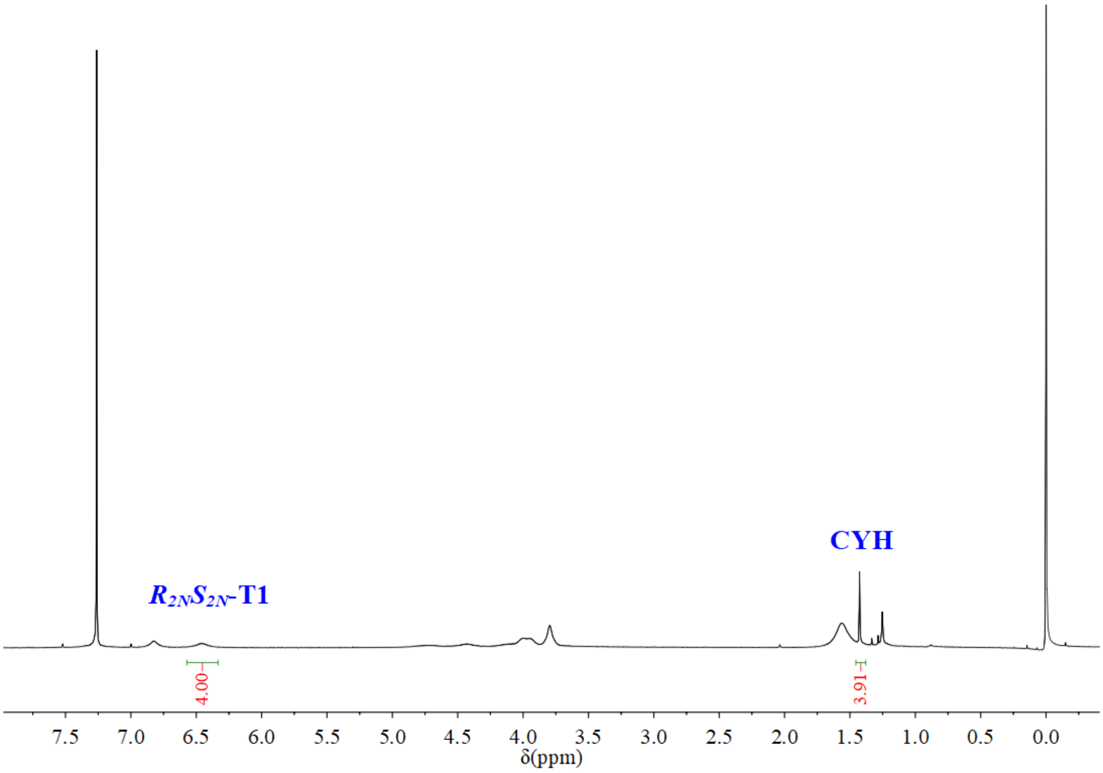

Figure S10. ^{1}H NMR spectrum (400 MHz, $CDCl_3$, 293 K) of ***$R_{2N}S_{2N}$*-T1** after adsorption of cyclohexane vapor for 10 hours.

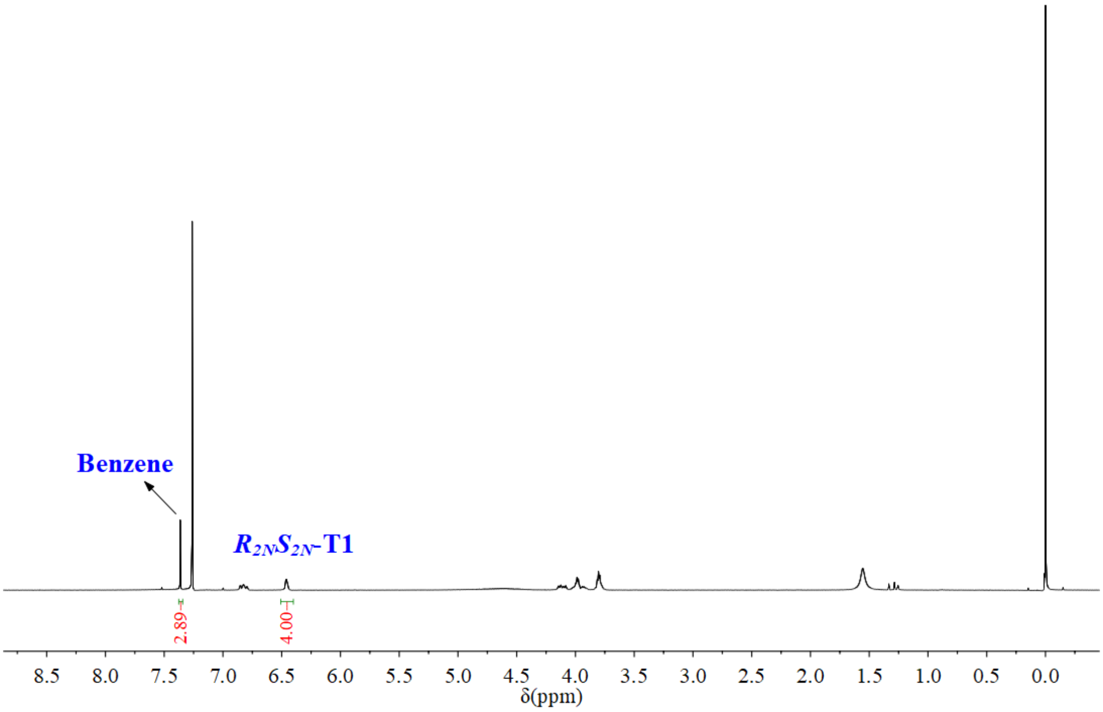

Figure S11. ^{1}H NMR spectrum (400 MHz, $CDCl_3$, 293 K) of ***$R_{2N}S_{2N}$*-T1** after adsorption of benzene vapor for 10 hours.

4. X-ray Crystallography Experimental, Data and Analysis

The crystal structures were determined by single-crystal X-ray analyses. Data collections were performed using a Bruker Apex Smart CCD diffractometer with Mo-Kα radiation with an φ–ω mode (λ = 0.71073 Å). The structures were solved with direct methods using the SHELXTL program and refined anisotropically with SHELXTL using full-matrix least-squares procedures. Crystallographic data and structural refinements parameters for all these crystals are given in **Table S1- S4.**

(i) Single crystals of ***rac*-T1** were obtained as color less plates by slow evaporation of the solution of **T1** in 1 : 1 (v/v) acetone and isopropyl ether at 25 ˚C.

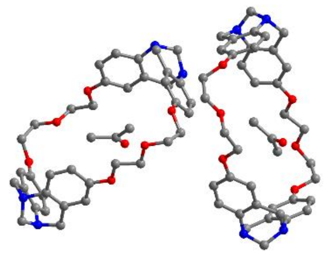

Figure S12. View of ***rac*-T1**

Table S1. Crystal data and structure refinement for ***rac*-T1**

Identification code	***rac*-T1**	
Empirical formula	$C_{41} H_{46} N_4 O_7$	
Formula weight	706.82	
Temperature	296(2) K	
Wavelength	0.71073 Å	
Crystal system	Triclinic	
Space group	P1	
Unit cell dimensions	a = 9.1047(6) Å	α = 71.973(2)°.
	b = 12.3823(8) Å	β = 89.804(2)°.
	c = 17.3387(12) Å	γ = 82.862(2)°.
Volume	1843.1(2) Å^3	
Z	2	
Density (calculated)	1.274 Mg/m^3	
Absorption coefficient	0.087 mm^{-1}	
F(000)	752	
Crystal size	0.280 x 0.250 x 0.220 mm^3	
Theta range for data collection	2.256 to 25.009°.	
Index ranges	-10<=h<=10, -12<=k<=14, -20<=l<=20	
Reflections collected	13545	
Independent reflections	9116 [R(int) = 0.0302]	
Completeness to theta = 25.009°	99.4 %	
Refinement method	Full-matrix least-squares on F^2	
Data / restraints / parameters	9116 / 3 / 941	

Goodness-of-fit on F^2	1.012
Final R indices [I>2sigma(I)]	R1 = 0.0487, wR2 = 0.1178
R indices (all data)	R1 = 0.0642, wR2 = 0.1276
Absolute structure parameter	-0.4(6)
Extinction coefficient	n/a
Largest diff. peak and hole	0.271 and -0.236 e.Å^{-3}

(ii) Single crystals of ***R**$_{2N}$**S**$_{2N}$***-T1** were obtained as color less plates by By slow evaporation of the solution of ***R**$_{2N}$**S**$_{2N}$***-T1** in 1 : 1 (v/v) dichloromethane and isopropyl ether at 25 ˚C.

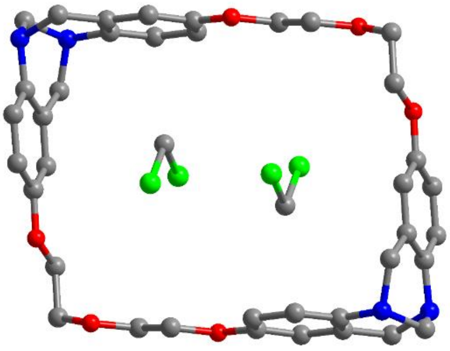

Figure S13. View of ***R**$_{2N}$**S**$_{2N}$***-T1**.

Table S2. Crystal data and structure refinement for ***R**$_{2N}$**S**$_{2N}$***-T1**

Identification code	***R**$_{2N}$**S**$_{2N}$***-T1**	
Empirical formula	$C_{40} H_{44} Cl_4 N_4 O_6$	
Formula weight	818.59	
Temperature	153(2) K	
Wavelength	0.71073 Å	
Crystal system	Monoclinic	
Space group	$P2_1/c$	
Unit cell dimensions	a = 14.642(4) Å	α = 90°.
	b = 8.452(2) Å	β = 99.638(4)°.
	c = 15.525(4) Å	γ = 90°.
Volume	1894.1(8) Å^3	
Z	2	
Density (calculated)	1.435 Mg/m^3	
Absorption coefficient	0.367 mm^{-1}	
F(000)	856	
Crystal size	0.260 x 0.220 x 0.190 mm^3	
Theta range for data collection	2.753 to 25.008°.	
Index ranges	-17<=h<=17, -10<=k<=9, -18<=l<=18	
Reflections collected	13732	
Independent reflections	3318 [R(int) = 0.0671]	
Completeness to theta = 25.008°	99.4 %	
Refinement method	Full-matrix least-squares on F^2	

Data / restraints / parameters	3318 / 0 / 244
Goodness-of-fit on F^2	1.037
Final R indices [I>2sigma(I)]	R1 = 0.0515, wR2 = 0.1445
R indices (all data)	R1 = 0.0695, wR2 = 0.1632
Extinction coefficient	n/a
Largest diff. peak and hole	0.691 and -0.713 e.Å^{-3}

(iii) Single crystals of ***S_{4N}*-T1.** were obtained as color less plates by slow evaporation of the solution of ***S_{4N}*-T1** in 1 : 1 (v/v) acetone and isopropyl ether at 25 ˚C.

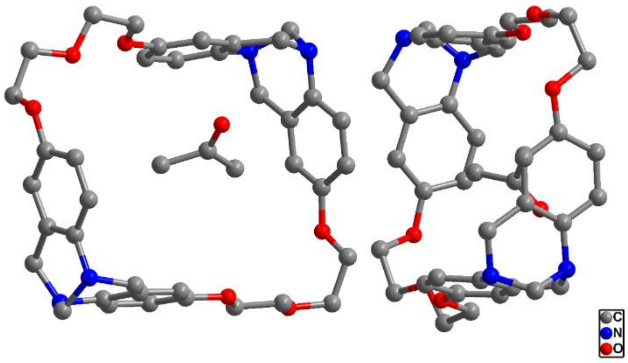

Figure S14. View of ***S_{4N}*-T1.**

Table S3. Crystal data and structure refinement for ***S_{4N}*-T1.**

Identification code	***S_{4N}*-T1.**	
Empirical formula	$C_{41} H_{46} N_4 O_7$	
Formula weight	706.82	
Temperature	153(2) K	
Wavelength	0.71073 Å	
Crystal system	Triclinic	
Space group	P1	
Unit cell dimensions	a = 9.0887(16) Å	α = 71.938(3)°.
	b = 12.347(2) Å	β = 89.794(3)°.
	c = 17.304(3) Å	γ = 82.857(4)°.
Volume	1830.6(6) Å^3	
Z	2	
Density (calculated)	1.282 Mg/m^3	
Absorption coefficient	0.088 mm^{-1}	
F(000)	752	
Crystal size	0.220 x 0.180 x 0.170 mm^3	
Theta range for data collection	1.750 to 25.009°.	
Index ranges	-9<=h<=10, -14<=k<=14, -20<=l<=20	
Reflections collected	13767	
Independent reflections	9788 [R(int) = 0.0406]	
Completeness to theta = 25.009°	98.4 %	

Refinement method	Full-matrix least-squares on F^2
Data / restraints / parameters	9788 / 3 / 941
Goodness-of-fit on F^2	1.029
Final R indices [I>2sigma(I)]	R1 = 0.0493, wR2 = 0.1143
R indices (all data)	R1 = 0.0598, wR2 = 0.1247
Absolute structure parameter	-0.2(9)
Extinction coefficient	n/a
Largest diff. peak and hole	0.207 and -0.219 e.Å^{-3}

(iv) Single crystals of **R_{4N}-T1.** were obtained as color less plates by slow evaporation of the solution of **R_{4N}-T1** in 1 : 1 (v/v) acetone and isopropyl ether at 25 ˚C.

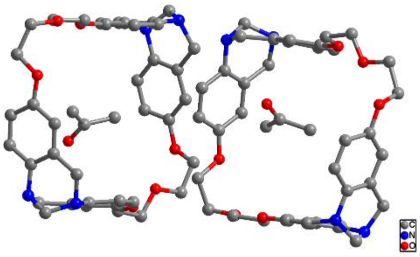

Figure S15. View of ***R_{4N}*-T1.**

Table S4. Crystal data and structure refinement for ***R_{4N}*-T1.**

Identification code	***R_{4N}*-T1.**	
Empirical formula	$C_{41} H_{46} N_4 O_7$	
Formula weight	706.82	
Temperature	296(2) K	
Wavelength	0.71073 Å	
Crystal system	Triclinic	
Space group	P1	
Unit cell dimensions	a = 9.1047(6) Å	α= 71.973(2)°.
	b = 12.3823(8) Å	β= 89.804(2)°.
	c = 17.3387(12) Å	γ= 82.862(2)°.
Volume	1843.1(2) Å^3	
Z	2	
Density (calculated)	1.274 Mg/m^3	
Absorption coefficient	0.087 mm^{-1}	
F(000)	752	
Crystal size	0.280 x 0.250 x 0.220 mm^3	
Theta range for data collection	2.256 to 25.009°.	
Index ranges	-10<=h<=10, -12<=k<=14, -20<=l<=20	
Reflections collected	13545	
Independent reflections	9116 [R(int) = 0.0302]	
Completeness to theta = 25.009°	99.4 %	
Refinement method	Full-matrix least-squares on F^2	
Data / restraints / parameters	9116 / 3 / 941	

Goodness-of-fit on F^2	1.016
Final R indices [I>2sigma(I)]	R1 = 0.0485, wR2 = 0.1142
R indices (all data)	R1 = 0.0640, wR2 = 0.1234
Absolute structure parameter	-0.4(6)
Extinction coefficient	n/a
Largest diff. peak and hole	0.270 and -0.237 e.Å^{-3}

5. Computational Details

The vertical binding energies between two neighbouring macrocycles in the supramolecular helix was estimated as

$$E_{interaction} = E_{complex} + E_{BSSE} - \sum_{i=1}^{2} E_i,$$

where $E_{complex}$ is the energy of the complex compound and E_i is the energy of the *i*th molecule in the complex compound. The calculations of these interactions were performed with Gaussian16 using two neighbouring macrocycles taken from optimized structure (at PBE-D/DND using DMol). Their energies were calculated with M06-2X functional using 6-311++G(2d,2p) basis set, the BSSE correction and an empirical dispersion correction (D3) are included.

Table S5 XYZ data of the geometries for interactions calculation. (The electronic energies in a.u.)

Pairs in the crystal of ***rac*-T1**				Pairs in the crystal of ***$R_{2N}S_{2N}$*-T1**			
C(frag=1)	-1.17503	9.50099	13.18037	C(frag=1)	4.54248	7.46569	5.13342
C(frag=1)	-2.28909	10.61850	21.82360	C(frag=1)	4.30080	8.15413	6.33534
H(frag=1)	-1.39734	10.14546	22.28849	C(frag=1)	2.98508	8.29481	6.80044
C(frag=1)	-1.18424	12.38472	20.70941	C(frag=1)	1.90324	7.76302	6.08305
H(frag=1)	-0.28504	12.20588	21.33524	C(frag=1)	2.15390	7.06891	4.87593
H(frag=1)	-1.80323	13.15196	21.21574	C(frag=1)	3.46465	6.93132	4.41358
C(frag=1)	-0.77101	12.88422	19.34217	H(frag=1)	3.67427	6.35573	3.50694
H(frag=1)	-1.62296	12.78051	18.64980	C(frag=1)	1.00986	6.40829	4.13222
H(frag=1)	-0.48051	13.94988	19.40250	H(frag=1)	0.68221	7.01944	3.27025
C(frag=1)	0.48157	11.82279	17.55356	H(frag=1)	1.33378	5.43592	3.73612
C(frag=1)	1.35556	10.77089	17.23029	C(frag=1)	-0.44372	7.52237	5.62269
H(frag=1)	1.85842	10.23753	18.04124	H(frag=1)	-0.46267	8.27677	4.82085
C(frag=1)	1.53818	10.40579	15.90104	C(frag=1)	0.10546	5.21897	6.02828
H(frag=1)	2.22484	9.59305	15.64085	C(frag=1)	0.30455	5.56886	7.37739
C(frag=1)	0.84162	11.05556	14.86582	C(frag=1)	0.34821	7.02614	7.78925
C(frag=1)	-0.04044	12.09899	15.19600	H(frag=1)	-0.60259	7.33274	8.26445
C(frag=1)	-0.19004	12.50113	16.53147	H(frag=1)	1.16007	7.19825	8.51566
H(frag=1)	-0.86694	13.33186	16.74906	C(frag=1)	0.56256	4.56506	8.32595
C(frag=1)	-0.87812	12.75829	14.11612	H(frag=1)	0.73505	4.85466	9.36448
H(frag=1)	-0.47423	13.76363	13.88817	C(frag=1)	0.67390	3.22733	7.93505
C(frag=1)	0.47947	11.58680	12.55615	C(frag=1)	0.53152	2.88311	6.58090
H(frag=1)	1.09228	12.50325	12.58240	H(frag=1)	0.66074	1.83602	6.30229
H(frag=1)	0.52749	11.14339	11.54949	C(frag=1)	0.23904	3.87198	5.64962
C(frag=1)	0.32996	9.32320	13.25681	H(frag=1)	0.13640	3.59629	4.59763
H(frag=1)	0.59148	8.60676	14.05050	C(frag=1)	1.24616	2.49818	10.15393

H(frag=1)	0.70946	8.89884	12.31070	H(frag=1)	0.34698	2.90012	10.65942
N(frag=1)	-0.91027	11.96036	12.86953	H(frag=1)	2.05082	3.25258	10.20044
N(frag=1)	1.02436	10.61003	13.51009	C(frag=1)	1.65297	1.19292	10.81471
O(frag=1)	-1.93496	11.17408	20.55376	H(frag=1)	1.74321	1.33772	11.90801
O(frag=1)	0.35349	12.09370	18.90261	H(frag=1)	0.86341	0.44986	10.62607
C(frag=1)	-1.73682	10.78448	13.00676	C(frag=1)	4.02654	1.26558	10.77294
C(frag=1)	-2.02070	8.39699	13.29780	H(frag=1)	4.14275	1.06616	11.85645
C(frag=1)	-3.12968	10.92822	12.99409	H(frag=1)	3.97967	2.36111	10.61903
C(frag=1)	-3.41166	8.54801	13.28122	C(frag=1)	6.94169	7.73313	5.28565
H(frag=1)	-1.59669	7.39604	13.43273	H(frag=1)	6.94935	7.37190	6.32937
H(frag=1)	-3.56938	11.92344	12.86953	N(frag=1)	-0.16188	6.20835	5.01255
C(frag=1)	-3.97674	9.82550	13.13777	N(frag=1)	0.56926	7.89597	6.61333
O(frag=1)	-4.14141	7.37645	13.41200	O(frag=1)	0.94533	2.18436	8.78325
H(frag=1)	-5.06027	9.97808	13.12856	O(frag=1)	2.85084	0.61939	10.27433
C(frag=1)	-5.55818	7.48239	13.23126	O(frag=1)	5.78538	7.23174	4.59304
H(frag=1)	-5.79050	7.91827	12.24324	C(frag=1)	7.62199	0.97701	10.16601
H(frag=1)	-6.00924	8.12894	14.00790	C(frag=1)	7.86366	0.28857	8.96409
C(frag=1)	-6.12710	6.07659	13.33349	C(frag=1)	9.17938	0.14789	8.49899
H(frag=1)	-5.75395	5.44547	12.49744	C(frag=1)	10.26123	0.67968	9.21638
H(frag=1)	-5.79887	5.61539	14.28743	C(frag=1)	10.01056	1.37379	10.42350
O(frag=1)	-7.54074	6.21235	13.26073	C(frag=1)	8.69981	1.51138	10.88585
C(frag=1)	-8.22946	4.96468	13.35766	H(frag=1)	8.49019	2.08697	11.79250
H(frag=1)	-7.77592	4.21115	12.68325	C(frag=1)	11.15460	2.03441	11.16721
H(frag=1)	-9.25126	5.16580	12.99893	H(frag=1)	11.48225	1.42326	12.02918
C(frag=1)	-8.25342	4.40343	14.76981	H(frag=1)	10.83068	3.00678	11.56331
H(frag=1)	-8.72912	3.40856	14.77165	C(frag=1)	12.60818	0.92033	9.67674
H(frag=1)	-7.22120	4.30669	15.15249	H(frag=1)	12.62714	0.16593	10.47858
O(frag=1)	-9.00309	5.31214	15.59480	C(frag=1)	12.05900	3.22373	9.27115
C(frag=1)	-8.82369	5.22874	16.95675	C(frag=1)	11.85991	2.87384	7.92204
C(frag=1)	-8.01070	4.28883	17.61021	C(frag=1)	11.81626	1.41656	7.51018
C(frag=1)	-9.50100	6.19345	17.71267	H(frag=1)	12.76705	1.10996	7.03498
H(frag=1)	-7.48486	3.50253	17.06750	H(frag=1)	11.00439	1.24445	6.78377
C(frag=1)	-7.87171	4.34535	18.99955	C(frag=1)	11.60190	3.87764	6.97348
C(frag=1)	-9.35650	6.25708	19.09971	H(frag=1)	11.42941	3.58804	5.93496
H(frag=1)	-10.11491	6.93571	17.19069	C(frag=1)	11.49056	5.21537	7.36438
H(frag=1)	-7.23781	3.60847	19.50404	C(frag=1)	11.63295	5.55959	8.71853
C(frag=1)	-8.53053	5.32063	19.75731	H(frag=1)	11.50372	6.60668	8.99714
C(frag=1)	-10.05867	7.35096	19.87981	C(frag=1)	11.92542	4.57072	9.64981
N(frag=1)	-8.35882	5.34317	21.18696	H(frag=1)	12.02806	4.84641	10.70180
H(frag=1)	-9.97015	8.31498	19.35230	C(frag=1)	10.91830	5.94452	5.14550
H(frag=1)	-11.13723	7.13076	19.96040	H(frag=1)	11.81748	5.54258	4.64001
N(frag=1)	-9.49226	7.49695	21.23876	H(frag=1)	10.11364	5.19012	5.09899

C(frag=1)	-9.40599	6.14750	21.81854	C(frag=1)	10.51149	7.24978	4.48472
C(frag=1)	-7.06036	5.90425	21.61407	H(frag=1)	10.42126	7.10498	3.39142
C(frag=1)	-8.20448	8.13084	21.21873	H(frag=1)	11.30105	7.99284	4.67336
H(frag=1)	-10.37836	5.64989	21.68499	C(frag=1)	8.13793	7.17712	4.52649
H(frag=1)	-9.18676	6.22188	22.88991	H(frag=1)	8.02171	7.37654	3.44298
H(frag=1)	-6.26427	5.45795	20.99400	H(frag=1)	8.18479	6.08159	4.68040
H(frag=1)	-6.88279	5.60516	22.66679	C(frag=1)	5.22277	0.70957	10.01378
C(frag=1)	-7.02190	7.41581	21.47869	H(frag=1)	5.21511	1.07080	8.97006
C(frag=1)	-8.11927	9.49503	20.89315	N(frag=1)	12.32634	2.23435	10.28688
C(frag=1)	-5.79269	8.08854	21.51299	N(frag=1)	11.59521	0.54673	8.68610
C(frag=1)	-6.89301	10.15007	20.87680	O(frag=1)	11.21913	6.25834	6.51618
H(frag=1)	-9.02842	10.03824	20.62099	O(frag=1)	9.31363	7.82331	5.02510
H(frag=1)	-4.88362	7.51429	21.69950	O(frag=1)	6.37908	1.21096	10.70639
C(frag=1)	-5.72145	9.45012	21.21389	H(frag=1)	5.12393	8.58749	6.90647
H(frag=1)	-6.83494	11.20494	20.59682	H(frag=1)	2.79298	8.83039	7.73509
O(frag=1)	-4.55887	10.19151	21.19156	H(frag=1)	-1.42499	7.48707	6.12038
C(frag=1)	-3.35174	9.55502	21.65460	H(frag=1)	6.95197	8.84060	5.28274
H(frag=1)	-3.03764	8.78225	20.93276	H(frag=1)	7.04054	-0.14479	8.39296
H(frag=1)	-3.54178	9.09659	22.64239	H(frag=1)	9.37148	-0.38769	7.56434
H(frag=1)	-2.65766	11.41682	22.49732	H(frag=1)	13.58945	0.95563	9.17905
H(frag=1)	-1.91033	12.89647	14.47946	H(frag=1)	5.21249	-0.39790	10.01669
C(frag=2)	-0.41398	17.21670	21.25672	H(frag=2)	12.03648	5.17723	13.77010
C(frag=2)	-1.80481	17.06643	21.24014	C(frag=2)	13.01773	5.14186	13.27241
H(frag=2)	-2.22862	16.06532	21.10522	H(frag=2)	12.99948	4.38708	12.47057
C(frag=2)	1.73269	16.15210	21.30669	N(frag=2)	13.29994	6.45554	12.66227
H(frag=2)	1.96482	16.58704	22.29470	N(frag=2)	14.03090	4.76818	14.26305
H(frag=2)	2.18387	16.79763	20.53004	C(frag=2)	14.47132	6.25553	11.78194
C(frag=2)	2.30187	14.74568	21.20445	C(frag=2)	13.56765	7.44486	13.67800
H(frag=2)	1.92837	14.11496	22.04050	C(frag=2)	15.36532	4.90082	13.73277
H(frag=2)	1.97326	14.28428	20.25052	C(frag=2)	13.80962	5.63778	15.43896
C(frag=2)	4.40394	13.63346	21.18028	C(frag=2)	15.61551	5.59515	12.52564
H(frag=2)	3.95060	12.87998	21.85469	H(frag=2)	14.14388	5.64445	10.91997
H(frag=2)	5.42598	13.83453	21.53901	H(frag=2)	14.79530	7.22813	11.38584
C(frag=2)	4.42776	13.07259	19.76814	C(frag=2)	13.76642	7.09516	15.02710
H(frag=2)	4.90380	12.07783	19.76629	C(frag=2)	13.70094	8.79214	13.29934
H(frag=2)	3.39602	12.97628	19.38546	C(frag=2)	16.44712	4.36901	14.45016
C(frag=2)	4.99807	13.89802	17.58119	H(frag=2)	12.85883	5.33114	15.91416
C(frag=2)	4.18507	12.95829	16.92774	H(frag=2)	14.62171	5.46564	16.16538
H(frag=2)	3.65924	12.17201	17.47044	C(frag=2)	16.92646	5.73285	12.06330
C(frag=2)	4.04627	13.01438	15.53839	C(frag=2)	14.02411	8.09891	15.97567
H(frag=2)	3.41227	12.27784	15.03391	C(frag=2)	13.99315	9.78095	14.23061
C(frag=2)	4.70490	13.99009	14.78063	H(frag=2)	13.59819	9.06754	12.24735
C(frag=2)	5.53093	14.92664	15.43823	C(frag=2)	17.76224	4.50984	13.98506

C(frag=2)	5.67561	14.86315	16.82527	H(frag=2)	16.25481	3.83366	15.38480
H(frag=2)	6.28969	15.60498	17.34726	C(frag=2)	18.00475	5.19834	12.78313
C(frag=2)	6.23305	16.01982	14.65813	H(frag=2)	17.13631	6.30856	11.15665
H(frag=2)	6.14485	16.98388	15.18564	H(frag=2)	14.19660	7.80958	17.01419
H(frag=2)	7.31161	15.79970	14.57754	C(frag=2)	14.13542	9.43666	15.58477
C(frag=2)	5.58038	14.81659	12.71940	H(frag=2)	14.12232	10.82819	13.95201
H(frag=2)	6.55321	14.31921	12.85295	H(frag=2)	18.58576	4.07656	14.55618
H(frag=2)	5.36141	14.89066	11.64804	O(frag=2)	19.24746	5.43237	12.24276
C(frag=2)	3.23475	14.57319	12.92387	O(frag=2)	14.40734	10.47950	16.43297
H(frag=2)	2.43912	14.12766	13.54394	C(frag=2)	20.40313	4.93071	12.93536
H(frag=2)	3.05754	14.27476	11.87115	C(frag=2)	14.70827	10.16611	17.80364
C(frag=2)	3.19672	16.08543	13.05926	C(frag=2)	21.59941	5.48691	12.17620
C(frag=2)	1.96740	16.75742	13.02495	H(frag=2)	20.41075	5.29239	13.97909
H(frag=2)	1.05830	16.18385	12.83844	H(frag=2)	20.41380	3.82345	12.93246
C(frag=2)	4.37914	16.79975	13.31921	H(frag=2)	13.80857	9.76390	18.30914
H(frag=2)	-0.78782	17.45163	13.60519	H(frag=2)	15.51244	9.41125	17.85015
N(frag=2)	5.66672	16.16586	13.29918	C(frag=2)	15.11499	11.47093	18.46442
N(frag=2)	4.53348	14.01231	13.35099	H(frag=2)	21.48356	5.28741	11.09270
O(frag=2)	0.31622	16.04522	21.12594	H(frag=2)	21.64666	6.58227	12.33012
O(frag=2)	3.71513	14.88114	21.27722	O(frag=2)	22.77514	4.84079	12.67481
O(frag=2)	5.17755	13.98162	18.94314	H(frag=2)	15.20542	11.32622	19.55772
C(frag=2)	0.15134	18.49470	21.40017	H(frag=2)	14.32564	12.21422	18.27578
C(frag=2)	-0.69573	19.59742	21.54385	O(frag=2)	16.31299	12.04461	17.92405
H(frag=2)	1.23486	18.64728	21.40938	C(frag=2)	23.97313	5.41447	12.13444
C(frag=2)	-2.08859	19.45368	21.53118	C(frag=2)	17.48872	11.39849	18.42266
H(frag=2)	-0.25603	20.59264	21.66842	C(frag=2)	24.37985	6.71929	12.79522
C(frag=2)	-2.65061	18.17012	21.35757	H(frag=2)	23.88270	5.55918	11.04114
N(frag=2)	-2.91515	20.62923	21.66842	H(frag=2)	24.76249	4.67118	12.32308
C(frag=2)	-4.15543	17.99275	21.28113	C(frag=2)	18.68500	11.95469	17.66350
C(frag=2)	-2.94708	21.42784	20.42183	H(frag=2)	17.60457	11.59799	19.50616
C(frag=2)	-4.30501	20.25593	21.98179	H(frag=2)	17.44146	10.30313	18.26874
H(frag=2)	-4.41691	17.27598	20.48745	H(frag=2)	25.27955	7.12150	12.28973
H(frag=2)	-4.53469	17.56761	22.22724	H(frag=2)	23.57569	7.47415	12.74871
N(frag=2)	-4.84982	19.27891	21.02785	O(frag=2)	24.68079	6.40590	14.16590
C(frag=2)	-3.78500	20.76776	19.34194	H(frag=2)	18.67737	11.59301	16.61977
H(frag=2)	-1.91507	21.56567	20.05849	H(frag=2)	18.67432	13.06195	17.66641
H(frag=2)	-3.35118	22.43312	20.64978	O(frag=2)	19.84066	11.45303	18.35610
H(frag=2)	-4.91750	21.17262	21.95554	C(frag=2)	24.95270	7.44874	15.01410
H(frag=2)	-4.35309	19.81224	22.98845	C(frag=2)	21.08338	11.68706	17.81573
C(frag=2)	-4.66726	19.72486	19.67212	C(frag=2)	25.06401	8.78649	14.62320
C(frag=2)	-3.63515	21.17001	18.00647	C(frag=2)	25.09497	7.10445	16.36825
C(frag=2)	-5.36331	19.07467	18.63690	C(frag=2)	21.32589	12.37556	16.61380

C(frag=2)	-4.30679	20.49243	16.98438	C(frag=2)	22.16166	11.15255	18.53557
H(frag=2)	-2.95856	22.00139	17.78888	C(frag=2)	25.32171	9.79024	15.57176
C(frag=2)	-5.18102	19.43981	17.30765	H(frag=2)	24.89153	9.07582	13.58467
H(frag=2)	-6.05015	18.26236	18.89709	H(frag=2)	24.96581	6.05722	16.64685
O(frag=2)	-4.17882	20.76343	15.63533	C(frag=2)	25.38719	8.09326	17.29953
H(frag=2)	-5.68377	18.90683	16.49670	H(frag=2)	20.50237	12.80884	16.04268
C(frag=2)	-3.05443	21.55388	15.19577	C(frag=2)	22.64100	12.51639	16.14870
C(frag=2)	-2.64113	21.05437	13.82853	C(frag=2)	23.47261	11.29025	18.07322
H(frag=2)	-2.20231	21.44951	15.88814	H(frag=2)	21.95181	10.57685	19.44221
H(frag=2)	-3.34514	22.61864	15.13545	C(frag=2)	25.52048	9.44054	16.92087
H(frag=2)	-3.54029	20.87457	13.20270	C(frag=2)	25.27850	11.24762	15.15990
H(frag=2)	-2.02211	21.82124	13.32220	H(frag=2)	25.48993	7.81786	18.35151
O(frag=2)	-1.89036	19.84311	13.98418	H(frag=2)	22.83331	13.05174	15.21406
C(frag=2)	-1.53636	19.28724	12.71434	C(frag=2)	23.72280	11.98458	16.86609
C(frag=2)	-0.47366	18.22422	12.88334	C(frag=2)	24.61681	10.62987	18.81692
H(frag=2)	-1.16775	20.08602	12.04062	N(frag=2)	25.78819	10.42986	17.93659
H(frag=2)	-2.42785	18.81459	12.24946	H(frag=2)	26.22930	11.55426	14.68470
H(frag=2)	-0.28363	17.76579	11.89556	H(frag=2)	24.46641	11.41977	14.43348
O(frag=2)	0.73346	18.86071	13.34638	N(frag=2)	25.05723	12.11722	16.33581
C(frag=2)	1.89604	18.11932	13.32405	H(frag=2)	24.94424	11.24095	19.67889
C(frag=2)	3.06760	18.81927	13.66114	H(frag=2)	24.29282	9.65727	19.21303
H(frag=2)	3.00953	19.87414	13.94113	C(frag=2)	26.07040	11.74354	17.32645
C(frag=2)	4.29386	18.16423	13.64479	H(frag=2)	26.08864	12.49832	18.12830
H(frag=2)	5.20301	18.70744	13.91695	H(frag=2)	27.05128	11.70842	16.82876

For two neighbouring macrocycles in the crystal of ***rac*-T1**, the energy of the complex compound is -4284.398230 a.u., the energy of fragment 1 using fragment 2 as ghost atoms is -2142.192155 a.u. while the energy of fragment 2 using fragment 1 as ghost atoms is -2142.191919 a.u. The energies for two neighbouring macrocycles in the crystal of **$R_{2N}S_{2N}$-T1** are -4284.386337 a.u., -2142.190666 a.u. and -2142.190725 a.u., correspondingly.

6. References

Brahim, A. A., Matsumoto, M., Miyahara, Y., Izumi, K., Suenaga, M., Shimizu, N., and Inazu, T. (1998). Synthesis and properties of a new series of Trögerophanes. *J. Heterocycl Chem. 35*, 209-213. doi: 10.1002/jhet.5570350139
